# A New *in Vitro* Anti-Tumor Polypeptide Isolated from *Arca inflata*

**DOI:** 10.3390/md11124773

**Published:** 2013-12-02

**Authors:** Jian Xu, Zhiyan Chen, Liyan Song, Lili Chen, Jianhua Zhu, Shuangshuang Lv, Rongmin Yu

**Affiliations:** 1 Biotechnological Institute of Chinese Materia Medica, Jinan University, Guangzhou 510632, China; E-Mails: lenovo1688@126.com (J.X.); chennybest@163.com (L.C.); tzhujh@jnu.edu.cn (J.Z.); lss313779851@sina.com (S.L.); 2 Department of Pharmacology, Jinan University, Guangzhou 510632, China; E-Mail: nihaochenzhi@126.com

**Keywords:** *Arca inflata* Reeve, polypeptide, structure analysis, anti-tumor activity

## Abstract

A new *in vitro* anti-tumor polypeptide, coded as J2-C3, was isolated from *Arca inflata* Reeve and purified by diethyl-aminoethanol (DEAE)-sepharose Fast Flow anion exchange and phenyl sepharose CL-4B hydrophobic chromatography. J2-C3 was identified to be a homogeneous compound by native polyacrylamide gel electrophoresis (Native-PAGE). The purity of J2-C3 was over 99% in reversed phase-high performance liquid chromatography (RP-HPLC). The molecular weight was determined as 20,538.0 Da by electrospray-ionization mass spectrometry (ESI-MS/MS). J2-C3 was rich in Glx (Gln + Glu), Lys, and Asx (Asp + Asn) according to amino acid analysis. Four partial amino acid sequences of this peptide were determined as L/ISMEDVEESR, KNGMHSI/LDVNHDGR, AMKI/LI/LNPKKGI/LVPR and AMGAHKPPKGNEL/IGHR via MALDI-TOF/TOF-MS and *de novo* sequencing. Secondary structural analysis by CD spectroscopy revealed that J2-C3 had the α-helix (45.2%), β-sheet (2.9%), β-turn (26.0%) and random coil (25.9%). The anti-tumor effect of J2-C3 against human tumor cells was measured by 3-(4,5-dimethylthiazol-2-yl)-2,5-diphenyltetrazolium bromide (MTT) assay, and the IC_50_ values of J2-C3 were 65.57, 93.33 and 122.95 µg/mL against A549, HT-29 and HepG2 cell lines, respectively. Therefore, J2-C3 might be developed as a potential anti-tumor agent.

## 1. Introduction

The significance of research and development of anti-tumor agents with high efficiency and low toxicity has drawn much attention globally. Over the past few decades, inspired by the immensity of the oceans and an almost incomprehensible level of biodiversity and chemical variation in the halobios, many researchers have enthusiastically pursued the discovery of novel anti-tumor medicine from marine organisms. To date, more than 30 new experimental anti-tumor agents derived from marine sources have entered clinical trials [1], including bryostatin-1, aplidine, ecteinascidin-743 (ET-743) [2], Kahalalide F [3], as well as derivatives of dolastatin such as TZT-1027 [4] and LU 103793 [5]. Interestingly, the ratio of compounds with anti-tumor activity in marine animals (1.8%) was much higher than that in microorganisms (0.3%), marine plants (0.1%), land plants (0.35%) and terrestrial animals (0.25%) [6]. Furthermore, increasing compounds especially proteins and polypeptides with anti-tumor activity have been found in marine animals [7,8], such as anthoplerintoxin, katsutoxin, snake venom, melittin, dolastatins and didemnins [9,10]. Therefore, marine animals have been an important resource for anti-neoplastic agents.

*Arca inflata* Reeve (*Scapharca broughtonii*) is a marine invertebrate that belongs to Arcidae family under Phylum Mollusca, Class Lamellibranchiata. This marine organism is widely distributed in China, Japan, Korea, *etc.* Some constituents, such as anticoagulant protein [11], aspartate racemase [12], Cu-Zn superoxide dismutase [13], were purified and characterized from *A.* *inflate*. Twelve novel microsatellite loci were also obtained and their genetic diversity and population genetics were evaluated [14]. A big defensin (Sb-BDef1) involved in immune response of Gram-negative microbial infection was identified from *A. inflata* [15]. However, no information is currently available on its anti-tumor activity either *in vitro* or *in vivo*. In our previous reports, two proteins with the *in vitro* anti-tumor activity were isolated from *Arca subcrenata*, a marine animal which also belongs to Arca genus of Arcidae family [16]. We also reported that a polypeptide fraction extracted from *A. subcrenata* showed the anti-tumor effect *in vitro* and *in vivo* [17]. Recently, a new antiproliferative and antioxidant peptide was purified from *A. subcrenata* by our research group [18]. As part of our ongoing project to screen bioactive peptides from marine organisms, we investigated the properties and bioactivities of the polypeptides from *A. inflata*.

In the present paper, we are the first to report an anti-tumor polypeptide isolated from *A. inflata*. The work will be beneficial to the research and development of a potential anti-tumor agent from this marine organism.

## 2. Results and Discussion

### 2.1. *In Vitro* Bioassay-Guided Isolation

Crude proteins of *A. inflata* were extracted by ammonium sulfate saturation and then subjected to the antiproliferative assay against tumor cells. The results showed that crude proteins suppressed the proliferation of A549, HepG2, K562 and HT-29 cells with the IC_50_ values less than 1000 μg/mL (Table 1).

**Table 1:** Antiproliferative activities of protein samples against six tumor cell lines (IC_50_, μg/mL ± SD, *n* = 3).

	IC_50_ (µg/mL)					
	A549	HepG2	NCI-H1650	SPC-A-1	K562	HT-29
Crude Proteins	699.76 ± 197.18	231.39 ± 29.75	>1000	>1000	571.18 ± 218.99	307.30 ± 32.83
J1	>1000	579.64 ± 201.55	>1000	672.98 ± 17.13	>1000	494.90 ± 130.47
J2	158.12 ± 48.21	226.16 ± 15.17	337.52 ± 86.69	897.55 ± 240.18	445.35 ± 94.48	423.64 ± 105.35
J3	>1000	>1000	>1000	>1000	>1000	>1000
J4	>1000	>1000	>1000	>1000	>1000	>1000
J2-C1	>1000	>1000	>1000	>1000	>1000	>1000
J2-C2	>1000	>1000	>1000	>1000	>1000	>1000
J2-C3	65.57 ± 2.53	122.95 ± 62.05	238.76 ± 158.47	172.78 ± 35.19	377.03 ± 74.97	93.33 ± 7.21
Cisplatin	0.71 ± 0.05	1.83 ± 0.81	7.02 ± 0.78	1.03 ± 0.08	13.05 ± 2.28	11.25 ± 1.09

**Figure 1:**
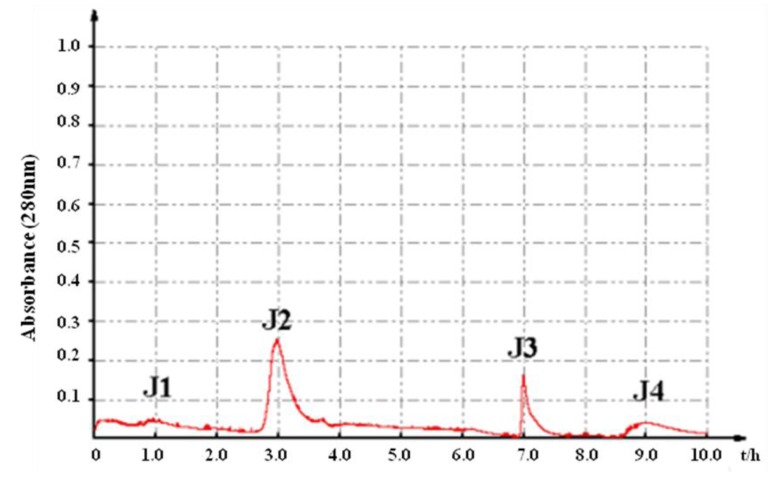
Purification of the crude proteins of *A. inflata* by anion exchange chromatography on a DEAE-Sepharose Fast Flow column.

In order to screen the anti-tumor components, the crude proteins were further purified by anion exchange chromatography monitored by spectrophotometry at 280 nm. Four fractions, J1, J2, J3 and J4, were obtained with increasing concentrations of NaCl on a DEAE sepharose column (Figure 1). The results demonstrated that fraction J2 exhibited the significant inhibition on the tumor cells’ proliferation. Therefore, the fraction J2 was further purified in order to get the active constituents.

**Figure 2:**
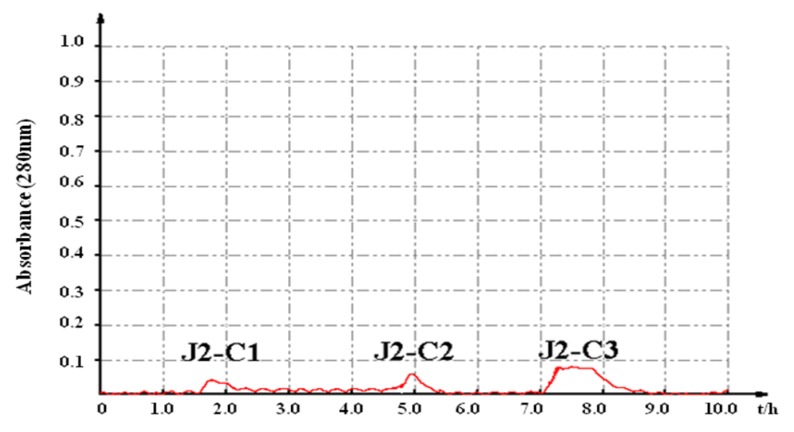
Purification of fraction J2 by phenyl sepharose CL-4B hydrophobic chromatography.

Fraction J2 was subjected to phenyl sepharose CL-4B hydrophobic chromatography. Three polypeptide compounds, namely J2-C1, J2-C2 and J2-C3, were obtained (Figure 2). The evaluation results of the *in vitro* anti-tumor activity suggested that J2-C3 had the strongest antiproliferative activity among all the fractions, with the IC_50_ values of 65.57 and 93.33 μg/mL against A549 and HT-29 cells, respectively (Table 1). Therefore, the further investigation of J2-C3 was warranted.

### 2.2. Characterization of Pure Peptide

**Figure 3:**
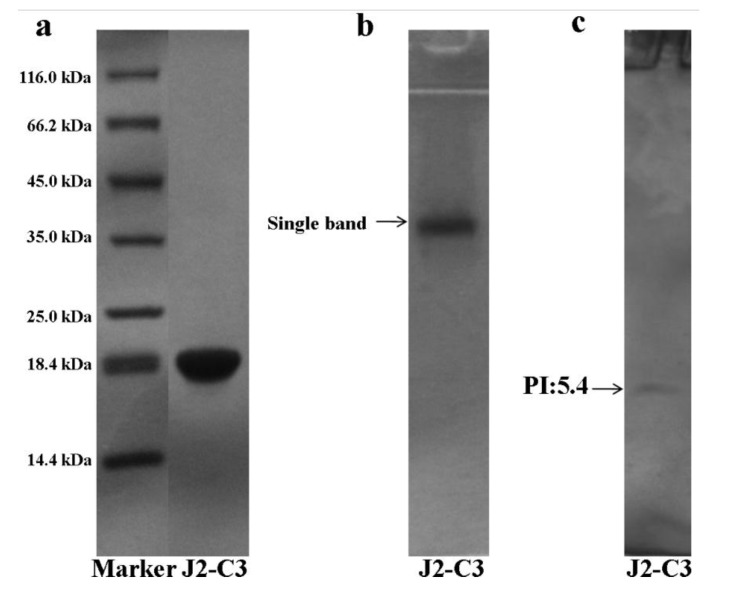
Electrophoresis. Purity and molecular weight analysis of J2-C3 by SDS-PAGE: (**a**) Lane 1, molecular weight marker; Lane 2, J2-C3. (**b**) Native-PAGE of J2-C3: Lane 1, J2-C3. (**c**) Isoelectric point determination of J2-C3 by IEF-PAGE: Lane 1, J2-C3.

**Figure 4:**
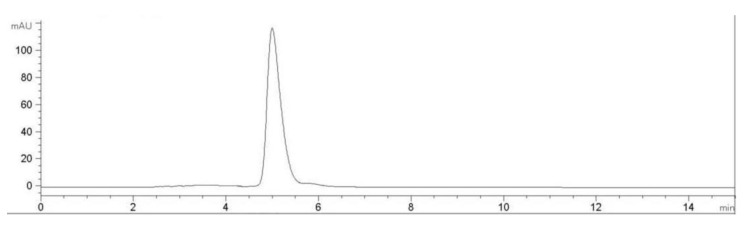
RP-HPLC profile of J2-C3. Performed on an Agilent 1100 HPLC system fitted with a ZORBAX^®^300SB-C8, Agilent column (5 μm, 300 Å, 4.6 × 250 mm).

The subunit constitution of J2-C3 was determined by sodium dodecyl sulfate polyacrylamide gel electrophoresis (SDS-PAGE) (Figure 3a). SDS-PAGE analysis revealed that J2-C3 was a pure polypeptide with an approximate molecular weight of 20 kDa. J2-C3 showed a single band in Native-PAGE, indicating that J2-C3 was homogeneous (Figure 3b). Furthermore, J2-C3 showed a single peak in RP-HPLC (Figure 4) and the purity was more than 99%. According to calibration curves, isoelectric focusing-polyacrylamide gel electrophoresis (IEF-PAGE) showed that J2-C3 was a single band and isoelectric point was 5.4 (Figure 3c).

**Figure 5:**
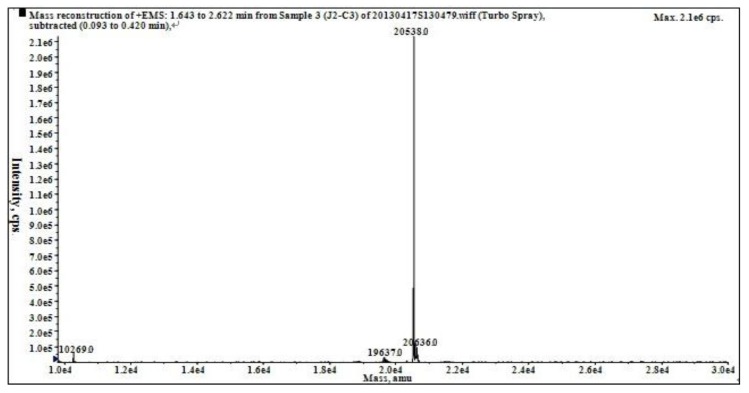
Mass spectrum of J2-C3.

The accurate molecular weight was 20,538.0 Da, as determined by ESI-MS/MS (Figure 5).

According to the modified version of the colorimetric phenol–sulfuric acid method [19], J2-C3 was not a glycopeptide because there were not any saccharides in this compound. The amino acid composition of J2-C3 is shown in Table 2. There were 16 kinds of amino acids existing in J2-C3, including high content for Glx (Gln + Glu), Lys, and Asx (Asp + Asn).

Matrix-assisted laser desorption/ionization time-of-flight mass spectrometry (MALDI-TOF-MS) of J2-C3 was measured with different ion signals in the mass range of 1200–2800 Da. The four precursor ions (*m/z* 1210.65, 1567.89, 1694.10 and 1716.04, respectively) were further detected by MS/MS analysis. Based on the manual calculation of the molecular weights and the *m/z* values, the amino acid sequence for each peptide was obtained. The sequence of fragment ion *m/z* 1210.65 was L/ISMEDVEESR, fragment *m/z* 1567.89 sequence was KNGMHSI/LDVNHDGR, fragment *m/z* 1694.10 with the amino acid sequence: AMKI/LI/LNPKKGI/LVPR, fragment *m/z* 1716.04 with the amino acid sequence: AMGAHKPPKGNEL/IGHR. The amino acid sequences were performed with the National Center for Biotechnology Information Basic Local Alignment Search Tool (NCBI BLAST) database and the low similarity of the amino acid sequences indicated that J2-C3 was a novel peptide.

**Table 2:** The amino acid composition of J2-C3 (g/100 g).

Amino Acid	J2-C3
Asx (Asp + Asn)	16.60
Glx (Glu + Gln)	12.00
Thr	3.89
Ser	5.10
Gly	2.95
Ala	4.76
Val	4.51
Met	1.87
Ile	5.37
Leu	8.03
Tyr	3.08
Phe	9.73
His	2.06
Lys	10.80
Arg	3.02
Pro	1.42

**Figure 6:**
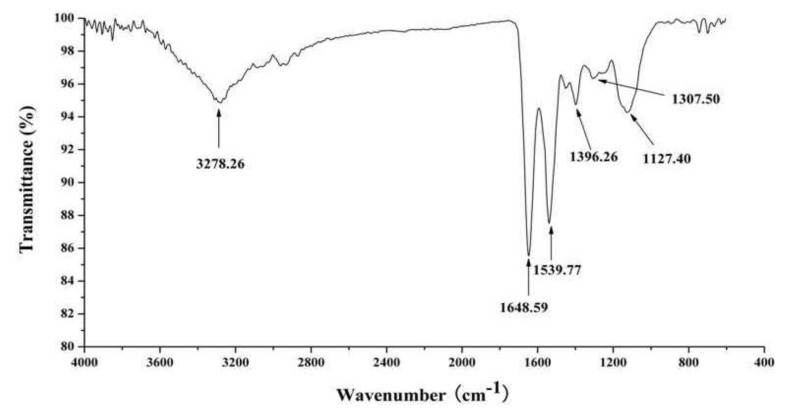
FT-IR spectrum of J2-C3.

The FT-IR spectrum for J2-C3 is shown in Figure 6. The spectrum contained a broad weaker peak around 3278.26 cm^−1^, which is characteristic of hydroxyl stretching vibration. Two relatively strong peaks in absorption at 1648.59 and 1539.77 cm^−1^ are characteristic of amide I and amide II, respectively, and the absorption at 1307.50 cm^−1^ is characteristic of amide III. The absorption at 1396.26 cm^−1^ should be assigned to the stretching vibration of C–N in the amide. Therefore, the observations provide further confirmation that J2-C3 is a peptide.

**Figure 7:**
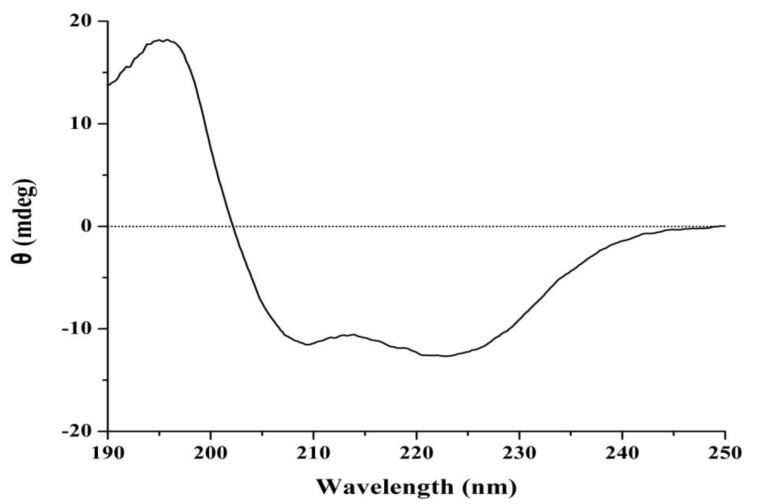
Far-UV circular dichroism spectrum of J2-C3.

It is well known that the far-UV CD spectrum of a protein is extremely sensitive toward secondary structure. As illustrated by Figure 7, the spectrum of J2-C3 showed two negative minima at 209 and 223 nm, a strong positive band at 197 nm and a broad shoulder that extended from 208 to 240 nm. The results demonstrated that there are α-helix of 45.2%, β-sheet of 2.9%, β-turn of 26.0% and random coil structures of 25.9% for J2-C3 by Jasco protein secondary structure estimation program. The total content of α-helix, β-sheet and β-turn accounted for more than 70% of the secondary structure; therefore, J2-C3 could be proposed as a polypeptide with highly ordered and stable conformation.

### 2.3. *In Vitro* Anti-Tumor Activity of J2-C3

**Figure 8:**
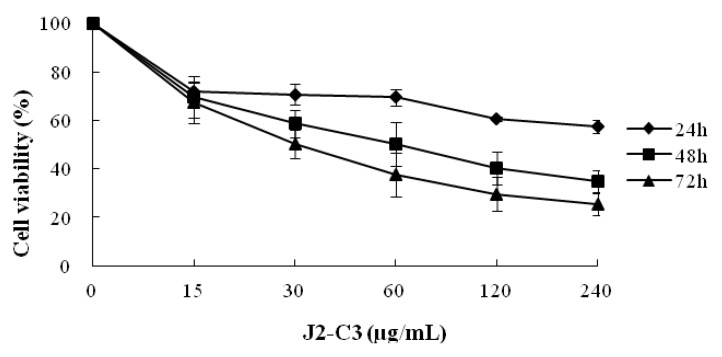
Inhibitory effect of J2-C3 on the proliferation of A549 cells.

The inhibitory effect of J2-C3 on the proliferation of A549 cells is shown in Figure 8. With the increase of concentration of J2-C3, the inhibitory effect of J2-C3 on the proliferation of A549 cells dramatically increased. Similarly, the inhibition rate clearly increased with the incubation time extending. Therefore, the inhibitory effect of J2-C3 on the proliferation of A549 cells existed in a time- and concentration-dependent manner. The IC_50_ values of J2-C3 were 63.86 and 35.19 µg/mL in 48 h and 72 h, respectively.

## 3. Discussion

Cancer is one of the most lethal diseases in human beings. Marine organisms are of interest to find new anti-tumor compounds due to their accessibility and therapeutic applications for many other diseases, and identification and isolation of some compounds with anti-tumor activity were reported [20,21,22,23,24].

We screened for the anti-tumor components in the protein extraction of the *A. inflate* through bioassay-guided isolation. As all the procedures of extraction and separation were conducted at the low temperature, it was possible to obtain natural product peptides [25,26]. A peptide, J2-C3, possessing the potent anti-tumor activity *in vitro* was purified by a combination of ammonium sulfate fractionation, anion exchange chromatography and hydrophobic chromatography. SDS-PAGE, Native-PAGE, IEF-PAGE, HPLC and carbohydrate concentration assay demonstrated that it was a homogeneous and no subunit non-glycopeptides, and its isoelectric point was 5.4.

According to the MS, J2-C3 has similar molecular size to polypeptide H3 which was isolated by our research group from *A. subcrenata* with strong anti-tumor activity *in vitro* [9], but smaller in size than the both anti-tumor proteins from *Syngnathus acus* [10] and *Aplysia dactylomela* [21]*.* Therefore, the biological function might not be directly related to the molecular weight.

The amino acid composition of J2-C3 was analyzed as shown in Table 2. It was rich in Glx (Gln + Glu), Lys, and Asx (Asp + Asn). Scientists designed a rich in Lysine polypeptide cationic micelle, which allowed for electrostatic interaction with the negatively charged siRNA and synergistic tumor growth inhibition *in vivo* [27]. Pokrovsky reported that L-Lysine α-oxidase showed considerable cytotoxicity against seven tumor cell lines [28]. El-Sersy also isolated a poly-lysine component which inhibited three human cell lines (Hela S3, HepG2 and CaCo) [29]. Our investigation demonstrated that J2-C3 had a higher level of lysine, which might be related to the anti-tumor activity, but its mechanism needs to be studied further.

The amino acid sequence had a slight similarity to other peptides from the BLAST search of NCBI database. Thus, J2-C3 could be proposed as a novel peptide.

Three relative strong peaks at 1648.59, 1539.77 and 1307.50 cm^−1^ for FT-IR spectrum of J2-C3 were characteristic absorptions of amide I, amide II and amide III, respectively, and the absorption at 1396.26 cm^−1^ should be assigned to the stretching vibration of C–N in the amide. All the data confirmed further that J2-C3 is a pure peptide. The characteristic absorptions at 1539.77cm^−1^ and 1396.26 cm^−1^ indicated that J2-C3 has higher account of α-helix [30]. The far-UV CD spectrum also showed that J2-C3 contained 45.2% α-helix, 2.9% β-sheet, 26.0% β-turn and 25.9% random coil structures by Jasco protein secondary structure estimation program and the total content of α-helix, β-sheet, and β-turn accounted for more than 70% of the secondary structure. Therefore, J2-C3 could be considered as a polypeptide with highly ordered and stable conformation. This result was similar with anti-tumor protein TBWSP31 from Chinese Tartary Buckwheat [26], which also had α-helix, β-sheet, and β-turn accounted for approximately 70%, and the IC_50_ value was 43.37 µg/mL against Bcap37 cell line.

The *in vitro* cytotoxicity assay demonstrated that J2-C3 possessed strong, selective antineoplastic properties. The IC_50_ values were 65.57, 93.33 and 122.95 µg/mL against A549, HT-29 and HepG2 cell lines, respectively. Taken together, J2-C3 showed higher anti-tumor activity and might be a potential agent for cancer treatment. Further study of the anti-tumor mechanism of J2-C3 is in progress.

## 4. Experimental Section

### 4.1. Materials

The samples of *A. inflata* were collected from Huangsha seafood market, Guangzhou, China. All of them were identified by Rongmin Yu (Jinan University, Guangzhou, China). The visceral mass was dissected, weighed and stored at −20 °C. DEAE Sepharose Fast Flow and Phenyl Sepharose CL-4B were purchased from GE Healthcare. Tris, SDS, coomassie brilliant blue R-250, Dimethyl sulfoxide (DMSO), MTT, Streptomycin, Penicillin and Cisplatin were obtained from Sigma Chemical Co. (St. Louis, MO, USA). RPMI-1640 and Fetal Bovine serum (FBS) were purchased from GIBCO Invitrogen Corporation (San Diego, CA, USA). Other commercially available chemicals and reagents were of analytical grade.

### 4.2. Preparation of Crude Protein

All extraction and separation procedures were carried out at 4 °C. Crude protein was extracted from *A. inflata* visceral mass (300 g) using phosphate buffer (pH 8.0, 30 mM) at a ratio of 1:3 (w/v) , a KQ-250B ultrasonic cleaner (Shanghai Yuzhen Co., Shanghai, China) with straight probe and continuous pulse was used to ultrasound for 40 min and then centrifuged at 10,000× *g* for 30 min. The supernatant was precipitated with 70%–100% saturation of solid ammonium sulfate [31]. After it was centrifuged (10,000× *g* for 30 min), the precipitate was re-dissolved in the 30 mM Tris-HCl buffer (pH 8.0), dialyzed against distilled water at 4 °C, and lyophilized.

### 4.3. Isolation of the Anti-Tumor Peptide

After lyophilization, the sample was applied to a DEAE-sepharose Fast Flow anion exchange column (2.5 cm × 40 cm). The column was pre-equilibrated with 30 mM Tris-HCl buffer (pH 8.0) and eluted at a flow rate of 1.0 mL/min. The column was stepwise eluted with 0, 0.1, 0.3 and 2 M NaCl prepared in the same buffer. The fraction with the highest anti-tumor activity was loaded onto a Phenyl Sepharose CL-4B hydrophobic chromatography column (2.5 × 40 cm) which had previously been equilibrated with 1.0 M (NH_4_)_2_SO_4_ prepared in 30 mM phosphate buffer (pH 8.0). A stepwise elution was carried out with decreasing concentrations of (NH_4_)_2_SO_4_ (1.0, 0.5 and 0 M) dissolved in 30 mM phosphate buffer (pH 8.0) at a flow rate of 1 mL/min. In each purification step, the elution was monitored at 280 nm by UV absorbance. Fractions or individual peaks were collected, dialyzed against distilled water and freeze-dried for assay of anti-tumor activity.

### 4.4. SDS-PAGE, Native-PAGE, IEF-PAGE

The fractions of eluted peptides were analyzed by sodium dodecyl sulfate polyacrylamide gel electrophoresis (SDS-PAGE) [32], using an acrylamide concentration of 5% for the stacking gels and 16% for the running gel. SDS-PAGE analysis under reducing conditions was used to check the purity of peptide and determine molecular weight. Protein bands were detected by the Coomassie blue staining method [33].

Native polyacrylamide gel electrophoresis (Native-PAGE) was carried out using the discontinuous system (17% separating and 4% stacking gel) [26]. The sample buffer was constituted of pH 8.8 Tris-HCl, 20% (v/v) glycerol, and 0.05% (w/v) bromophenol bule. Electrophoresis was conducted at a constant current of 30 mA for about 2 h. The gels were stained in Coomassie brilliant blue R-250.

Isoelectric focusing-polyacrylamide gel electrophoresis (IEF-PAGE) [18]: Ampholyte (40%, pH 3.5–10.0) was used to prepare IEF gel with acrylamide concentration of 5%. IEF-PAGE was carried out on BIO-RAD power PAC-300 and Mini-PROTEAN 3 cell provided by BIO-RADKWS at 150 V for 75 volt-hours (vh), then at 200 V for 500 vh. The IEF-PAGE gel unloaded samples was washed by double-distilled water, and sliced into pieces of 0.5 cm in length from acidic terminal to basic terminal, then separately dipped into Eppendorf tubes containing 2.0 mL of 10 mM KCl for 30 min. The pH value of the liquid around each slice was measured. The gel-loaded sample was fixed with 10% trichloroacetic acid for 30 min, and rinsed thoroughly with destaining solution (0.25% SDS, 33% ethanol, and 10% acetic acid). Data were derived from calibration curve of isoelectric point with the length of gel as abscissa and pH value as ordinate.

### 4.5. Reversed Phase-High Performance Liquid Chromatography

HPLC analysis was performed on an Agilent series 1100 HPLC system fitted with a reversed-phase high performance liquid chromatography (RP-HPLC) cartridge, 4.6 × 150 mm filled with ZORBAX^®^300SB-C8, 5 µm (Agilent, Foster City, CA, USA). The elution solvent system was composed of water-trifluoroacetic acid (solvent A; 100:0.1, v/v) and acetonitrile-trifluoroacetic acid (solvent B; 100:0.1, v/v). Gradient elution from 20% to 100% of solvent B in 15 min; flow rate: 1 mL/min; detection wavelength: 280 nm; column temperature 30 °C.

### 4.6. Carbohydrate Concentration Assay

The total neutral sugar content of the purified peptide was determined by the modified version of the colorimetric phenol-sulfuric acid method using glucose as standard. The absorbance at 490 nm was used to determine the amount of carbohydrate in the sample [19].

### 4.7. Molecular Weight Determination

To determine the precise molecular weight of the purified peptide, the sample was dissolved in water of HPLC grade and loaded into an API type 4000 QTRAP mass spectrometer (Applied Biosystem, Foster City, CA, USA). The sample was passed at a flow rate of 20 μL/min via the electrospray interface, which was operated in the positive electrospray ionization (ESI + ve) mode. The gas used for drying (35 psi) and ESI nebulization (45 psi) was high-purity nitrogen. Spectra were recorded over the mass/charge (*m/z*) range 10,000–25,000.

### 4.8. Amino Acid Analysis

The amino acid composition of the sample was determined with an automatic amino acid analyzer (Agilent 1100, Foster City, CA, USA). The samples were hydrolyzed with 6 mol/L HCl for 24 h at 110 °C in a sealed tube. Tryptophan was not determined.

### 4.9. MALDI-TOF-MS

The amino acid sequence of peptide was identified by the reference [34] with some modifications. J2-C3 was excised from SDS-PAGE gel and digested. Tryptic peptides were lyophilized and dissolved in 10 μL of a 50% acetonitrile/0.1% TFA solution. An amount of 0.4 μL of the sample was spotted onto the MALDI sample target plate, and then 0.4 μL of a saturated matrix solution of α-cyano-4-hydroxycinnamic acid prepared in 50% acetonitrile/0.1% TFA was added. Peptide mass spectra were obtained on a 4800 Proteomics Analyzer MALDI-TOF mass spectrometer (Applied Biosystems, Foster City, CA, USA) in the positive ion reflection mode. After an external calibration with a mixture of angiotensin II (Mr, 1046.54180), angiotensin I (Mr, 1296.68478), substance P (Mr, 1347.73543), bombesin (Mr, 1619.82235), ACTH clip 1–17 (Mr, 2093.0868), ACTH clip 18–39 (Mr, 2465.1990), Somatostatin 28 (Mr, 3147.4714), spectra were obtained in the mass range between 900 and 3500 Da with 500 laser shots. For each sample spot, a data-dependent acquisition method was created to select the four most intense peaks for subsequent MS/MS data acquisition, excluding those from the matrix, due to trypsin autolysis or acrylamide peaks. MS/MS spectra were acquired with 1200 laser shots in the mass range from 10 Da to the mass of parent ion using an interpretation method presented on instrument software, where the four most intense peaks were selected and MS/MS spectra were generated automatically. To ensure a reliable identification, the results from both the MS and MS/MS spectra were used in the database search. Peptide identification was accepted when the score read by the Mascot search routine was higher than 90. The sequence of peptide fragments was determined by *de novo* sequencing using the Applied Biosystems software as presented by Yergey *et al.* [35].

### 4.10. Infrared Spectroscopy

The sample was analyzed by infrared spectroscopy using a EQUINOX55 FT-IR spectrometer (Bruker, Bremen, Germany). The lyophilized sample was fully dried in a dryer containing P_2_O_5_, then mixed with potassium bromide powder and pressed into tablets. For sample, we recorded 100 scans between 4000 and 400 cm^−1^ at a resolution of 2 cm^−1^.

### 4.11. Circular Dichroism Spectroscopy

The concentration of J2-C3 was 0.01 mg/mL in distilled water, and the sample solution was filtered through a 0.02 µm membrane prior to circular dichroism (CD) analysis. CD measurement was carried out at 20 °C using a Jasco J-810 spectropolarimeter (Japan Spectroscopic Co., Ltd., Hachioji, Tokyo, Japan). Quartz cells with an optical path length of 0.1 cm were used. Each spectrum was recorded as the average of eight scans. The scan range was 250–190 nm, the scan speed was 50 nm/min, the data interval was 0.2 nm, the bandwidth was 2 nm, the sensitivity was 20 mdeg, and the response time was 0.5 s. CD spectra were corrected for solvent contributions and expressed in terms of specific ellipticities *versus* wavelength. The proportions of the secondary structure fractions (α-helix, β-sheet, β-turn, and random coil) were determined using the Jasco protein secondary structure estimation program (Japan Spectroscopic Co., Hachioji, Tokyo, Japan) based on the method of Yang and Chen [36,37]. Analyses were performed in triplicate.

### 4.12. *In Vitro* Cell Growth Inhibition Assay

#### 4.12.1. Cell Lines and Culture

Human lung adenocarcinoma cell lines (A549, NCI-H1650, SPC-A-1), human liver carcinoma cell line (HepG2), human chronic myeloid leukemia cell line (K562), and human colon carcinoma cell line (HT-29) were provided by the Shanghai Institutes for Biological Sciences, Chinese Academy of Sciences, China. All cells were cultured in RPMI-1640 medium supplemented with 10% heat-inactivated fetal bovine serum, 100 U/mL penicillin and 100 U/mL streptomycin and cultured in an atmosphere of 5% CO_2_ at 37 °C. Cells were collected for the experiments in the logarithmic growth phase.

#### 4.12.2. Cell Growth Inhibition Assay

For the anti-tumor screening tests, samples were dissolved and diluted to 3.9, 15.6, 62.5, 250 and 1000 μg/mL by RPMI-1640 medium. For the tests of time- and concentration-dependent effects, the concentrations of J2-C3 were 15, 30, 60, 120 and 240 μg/mL. The A549 cells were treated with different concentrations of J2-C3 for 24, 48 and 72 h. All tests were performed by MTT assay according to the reference [38] with some modification. Briefly, cells were seeded in the well of 96-well culture plates and incubated with different concentrations of samples for a setting time. After that, 20 μL of MTT (5 mg/mL) was added to each well, and then the plates were incubated for another 4 h at 37 °C. The supernatant was aspirated and MTT-formazan crystals were dissolved in 200 μL of DMSO. Absorbance was measured spectrophotometrically at 570 nm. Cell growth inhibition was evaluated by comparing the absorbance of treated and untreated cells. The percentage of cell growth inhibition was calculated as the following formula:

Inhibitory rate % = (A_570,control_ − A_570,sample_)/(A_570,control_ − A_570,blank_) × 100%

### 4.13. Statistical Analysis

All of the tests were conducted in triplicate and the experimental data were expressed as the mean ± standard deviation. GraphPad Prism 5.0 was used for statistical analysis.

## 5. Conclusions

In summary, this is first report documenting a new anti-tumor peptide from *A. inflata*, and it was purified by bioactive guideline isolation. It exhibited significant anti-tumor activity against A549, HT-29 and HepG2 cells, and displayed the *in vitro* anti-tumor activity against A549 cells in a time- and concentration-dependent manner. Further studies would focus on the elucidation of the action mechanism.
